# Dry eye disease in patients with type II diabetes mellitus: A retrospective, population-based cohort study in Taiwan

**DOI:** 10.3389/fmed.2022.980714

**Published:** 2022-08-23

**Authors:** Li-Yen Pan, Yu-Kai Kuo, Tien-Hsing Chen, Chi-Chin Sun

**Affiliations:** ^1^Department of Ophthalmology, Chang Gung Memorial Hospital, Taoyuan, Taiwan; ^2^Department of Ophthalmology, Chang Gung Memorial Hospital, Keelung, Taiwan; ^3^Department of Cardiology, Chang Gung Memorial Hospital, Keelung, Taiwan; ^4^School of Traditional Chinese Medicine, Chang Gung University, Taoyuan, Taiwan; ^5^Biostatistical Consultation Center of Chang Gung Memorial Hospital, Keelung, Taiwan; ^6^Department of Medicine, College of Medicine, Chang Gung University, Taoyuan, Taiwan

**Keywords:** hypoglycemic agent, dry eye disease, diabetes mellitus, risk and protective factors, SLGT-2 inhibitor, GLP-1 agonist, DPP4 inhibitor, insulin

## Abstract

**Purpose:**

To investigate the risk and protective factors of dry eye disease (DED) in patients with type II diabetes mellitus (DM).

**Design:**

A retrospective cohort study using Chang- Gung research database collecting data from 2005 to 2020.

**Methods:**

Patients with type II DM were included, and those with previous ocular diseases were excluded. Ten thousand twenty nine developed DED (DED group), and 142,491 didn't (non-DED group). The possible risk and protective factors were compared and analyzed using the logistic regression model.

**Results:**

A majority of the DED group were female with significantly higher initial and average glycated hemoglobin levels, and higher incidence of diabetic neuropathy and retinopathy. In conditional logistic regression model, advanced age was a risk factor. After adjusting for sex, age, and DM duration; average glycated hemoglobin level, diabetic neuropathy, retinopathy, and nephropathy with eGFR 30 ~ 59 and intravitreal injection, vitrectomy, pan-retinal photocoagulation, and cataract surgery were contributing factors of DED. Considering antihyperglycemic agents, DPP4 inhibitor, SGLT2 inhibitor, GLP-1 agonist, and insulin monotherapy and dual medications combining any two of the aforementioned agents were protective factors against DED compared with metformin alone. In the monotherapy group, SLGT2 inhibitor had the lowest odds ratio, followed by GLP1 agonist, DPP4 inhibitor, and insulin.

**Conclusions:**

DED in patients with DM is associated with female sex, advanced age, poor diabetic control, microvascular complications and receiving ocular procedures. GLP-1 agonist, SGLT-2 inhibitor, DPP4 inhibitor, and insulin are superior to metformin alone in preventing DM-related DED. A prospective randomized control trial is warranted to clarify our results.

## Introduction

Diabetes mellitus (DM) is becoming a global health issue, and the epidemic has continuously occurred over the past decades (1). According to the International Diabetes Federation, in 2021, 747,000 individuals died due to DM in southeast Asia alone, and >11% of the total population in southeast Asia will develop DM in 2045 (2). There are numerous DM-related microvascular and macrovascular comorbidities, including ocular complications. The occurrence of retinopathy, papillopathy, cataract, glaucoma, and ocular surface disease in patients with DM were well-investigated in previous studies (3–5). Given its major impact on vision, retinal disease, cataract, and glaucoma have been the major concerns of ophthalmologists (6, 7), whereas minor ocular surface diseases, such as dry eye disease (DED), were often overlooked, with a previous study showing that 51.3% of DM-related dry eye syndrome cases were underdiagnosed (8).

According to the definition given by the Tear Film & Ocular Surface Society, DED is a multifactorial disease caused by loss of tear film homeostasis and inflammation with neurosensory abnormalities potentially involved in the pathogenesis (9). In previous studies, abnormal tear dynamics was noted both *in vitro* and *in vivo* in individuals with DM with osmolarity changes (10–12). Altered enzyme metabolism and decrease mucin secretion may also contribute to DM-related DED (13). Moreover, lacrimal gland and lacrimal functional unit dysfunction (14, 15) caused by diabetic neuropathy plays an important role in DM-related DED; furthermore; the aforementioned pathology along with Meibomian gland dysfunction (8, 16) often leads to tear film instability (12) due to decreased quantity and quality of the tear lipid, resulting in DED.

In patients with DM, ocular sequelae can frequently develop and sometimes require surgical intervention. For instance, long-term hyperglycemia was associated with cataract formation (17), and cataract surgery will be indicated for visual improvement; moreover, the development of diabetic retinopathy might also require interventions at different stages, including pan-retinal photocoagulation (PRP) (18, 19), intravitreal injection (IVI) of anti-vascular endothelial growth factor or steroid (20), and even trans-pars plana vitrectomy (TPPV) (19). These ocular surgeries are associated with DED in previous studies, with the incidence of post-cataract DED of 9–32% (21, 22). Accumulating evidence also demonstrated that IVI was associated with deterioration of ocular surface health (20, 23); TPPV contributed to the development of signs and symptoms of DED, and PRP induced the decrease in tear break-up time and Schirmer test value (24).

Previous studies had investigated the prevalence of DED and relevant risk factors in patients with DM. The results regarding the prevalence of DED in patients with DM varied from studies, ranging from 15 to 54.3% (5, 25–27). Reported risk factors included but were not limited to advanced age, female sex, smoking, higher glycated hemoglobin (HbA1c) level, and DM retinopathy (3, 4, 16, 28, 29). Conflicting results regarding the role of diabetic neuropathy and nephropathy were reported among studies (26, 30, 31). However, the effects of antihyperglycemic medications on ocular surface disease are unclear. Therefore, this study aimed to investigate the associated factors of DED in a DM patient cohort, with specific focus on the effects of antihyperglycemic agents.

## Methods

### Data source

The study protocol was approved by the Institutional Review Board of Chang Gung Memorial Hospital (approval no.: 202001925B0C601) and followed the tenets of the Declaration of Helsinki. The Chang Gung Research Database (CGRD) contains multi-institutional standardized electronic medical records (EMR) from seven Chang Gung Memorial Hospital (CGMH) across the nation, including two medical centers, two regional hospitals, and three district hospitals. The EMR contains the patient-level data derived from electronic medical charts of patients established for administrative and healthcare purposes for CGMH. The Department of Information Systems Management of CGMH integrated and standardized all EMR from CGMH without selection criteria and established CGRD for research purposes. All data from CGRD are encrypted and de-identified, and the database contains laboratory data, inpatient data, outpatient data, emergency patient data, pathological data, nursing data, charge data, disease category data, and surgical data.

### Type II DM cohort

We included patients with type II DM from January 1, 2005, to December 31, 2020, using the International Classification of Disease, ninth version, Clinical Modification code (ICD-9-CM) 250.xy (x = 0, 1, 2, 3, 4, 5, 6, 7, 8, 9; y = 0, 2), and tenth version (ICD-10-CM) E11.xyza (x = 1, 2, 3, 4, 5, 6, 8, 9; y = 0, 1, 2, 3, 4, 5, 6, 7, 9; z = 0, 1, 2, 3, 4, 5, 8, 9; a = 1, 2, 3, 9). To ensure the diagnostic accuracy and for analysis of purpose, we only enrolled those who had HbA1c data, which we will brief as DM cohort in the study. The DM duration in our study is defined as the time from the day the patient was diagnosed to the last day of follow-up.

### DED and non-DED groups

We further identified patients with DED using ICD-9-CM 370.33, 372.53, 375.15, 710.2, and ICD-10-CM H16.22x (x = 1, 2, 3, 9), H04.12x (x = 1, 2, 3, 9), and for diagnostic accuracy, we excluded those who did not receive either lubricant and/or topical anti-inflammation agent after the diagnosis was established. Those with DED were classified as the DED group, and those without DED were classified as the non-DED group. We excluded those who had died during the study period to exclude serious health condition. Moreover, we excluded those who are aged <18 years and not using antihyperglycemic agents and those who had pre-existing DED before the development of DM, legal blindness, previous ocular evisceration surgery, glaucoma, uveitis, and congenital eye abnormality and had undergone cornea transplantation before the diagnosis of DED.

### DM comorbidity

Patients with comorbidities, including diabetic retinopathy, diabetic nephropathy, and diabetic neuropathy, which developed before the diagnosis of DED in the DED group and before the end of the follow-up in the non-DED group, were identified using ICD-9-CM and ICD-10-CM codes (Supplementary Table 1). For diabetic nephropathy, we further divided the patient into three groups based on the estimated glomerular filtration rate (eGFR) (≥60; 30–59; <30).

### Diabetic antihyperglycemic agents

In the subgroup analysis for diabetic medications, the medication group was defined using medication possession ratio (MPR). Those who were not receiving either metformin, dipeptidyl peptidase 4 (DPP-4) inhibitor, glucagon-like peptide-1 receptor (GLP-1) agonist, sodium-glucose cotransporter-2 (SGLT-2) inhibitor, and insulin or in those who are using these medications but did not reach our target MPR were categorized into group “other/non-routine medications.” In the monotherapy group, patients had received either one of the aforementioned medications, with MPR >50% throughout the whole course. We defined the dual therapy group as patients who had received any two medications of metformin, DPP4 inhibitor, GLP-1 agonist, SGLT-2 inhibitor, and insulin with the MPR >60% combined and >30% each throughout the follow-up period.

### Ocular procedures

In the subgroup analysis for the procedure received, we identified those who underwent IVI, TPPV, cataract surgery, and PRP before the diagnosis of DED in the DED group and before the end of the follow-up in the non-DED group using surgical coding in CGRD (Supplementary Table 2).

### Outcome measurements

Risk and protective factors for DED in the DM cohort were investigated, including basic demographic data, HbA1c levels (initial HbA1c level while the diagnosis was made, and average data throughout the follow-up course), microvascular and macrovascular comorbidities (including diabetic retinopathy, diabetic nephropathy, and diabetic neuropathy), antihyperglycemic agents (including metformin, DPP-4 inhibitor, GLP-1 agonist, SGLT-2 inhibitor, insulin, and dual therapy), and ocular procedures received (including IVI, cataract surgery, TPPV, and PRP). Comorbidities were collected according to the ICD-9-CM and ICD-10-CM code, procedure using operative codes in the CGMH group, and drugs using anatomical therapeutic chemical codes.

### Statistical analysis

All statistics were calculated using SPSS software version 23.0 for Windows (SPSS, Inc., Chicago, IL, USA). Continuous variables are presented as mean and standard deviation. Pearson's chi-square tests were used to compare sex, DM duration, comorbidity (nephropathy, neuropathy and retinopathy), antihyperglycemic agents, and procedures received between the DED and non-DED groups. Student's *t*-test was used to compare age, initial HbA1c level, and average HbA1c level between the two groups. The conditional logistic regression model was conducted to analyze the risk and protective factors for DED. A *P*-value <0.05 was deemed to be statistically significant. Moreover, adjusted odds ratio (OR) was used to determine the characteristic of each factor as either contributing (OR > 1) or protective factor (OR < 1).

## Results

**Figure 1:**
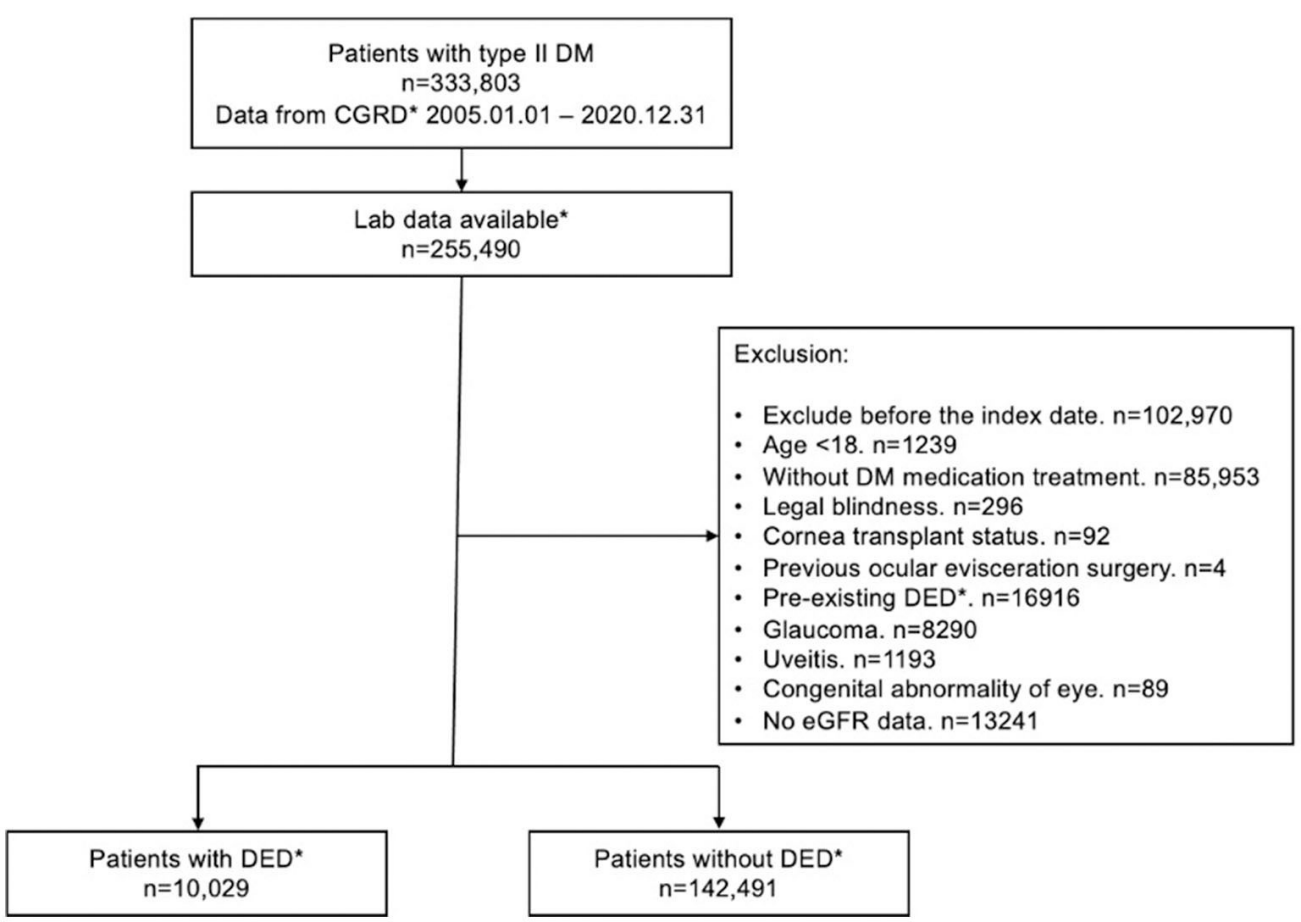
Flow chart for inclusion and exclusion of patients from the CGRD. CGRD, Chang Gung Research Database. DED, dry eye disease; Laboratory data available: with available HbA1c data.

Overall, 333,803 patients with type II DM were identified from January 1, 2005, to December 31, 2020, in the CGRD, and 255,490 of them had HbA1c data. We further excluded those who are aged <18 years (*n* = 1,239), without any antihyperglycemic agent use (*n* = 85,953), and those who had pre-existing DED before DM occurred (*n* = 16,916), legal blindness (*n* = 296), previous ocular evisceration surgery (*n* = 4), glaucoma (*n* = 8,290), uveitis (*n* = 1,193), and congenital eye abnormality (*n* = 89) and had underwent cornea transplantation (n = 92) before the diagnosis of DED or before the end of follow-up for the non-DED group. Moreover, those who did not have eGFR data were also excluded (*n* = 13,241). A total of 152,520 remained, with 10,029 of them having DED (DED group) and 142,291 did not develop DED throughout the follow-up course (non-DED group) (Figure 1). In the demographic data before age and sex matching, female predominance was noted in the DED group (*p* < 0.001) with younger age at the diagnosis of type II DM (*p* = 0.012). The HbA1c level was higher in the DED group in both initial data (*p* < 0.001) and average data (*p* < 0.001) (Table 1). After age and sex matching, the incidence of both retinopathy and neuropathy was higher in the DED group (*p* < 0.001, *p* < 0.001, respectively); however, the incidence of nephropathy was lower in the DED group but was not statistically significant (*p* = 0.835). However, when we further examined the distribution of different stages of nephropathy using eGFRs, those who had lower eGFR (30–59 group and <30 group), which indicated more severe forms of diabetic nephropathy, was significantly observed in the DED group (*p* < 0.001) (Table 2). In ocular procedures received, the percentage of all procedures, including IVI, TPPV, PRP, and cataract surgery, was significantly higher in the DED group compared with the non-DED group (*p* < 0.001, *p* < 0.001, *p* < 0.001, *p* < 0.001, respectively) (Table 3). In conditional logistic regression model, female sex was a contributing factor without statistical significance, and advanced age was a risk factor for DED (OR, 1.18, *p* < 0.001); however, longer DM duration (5–10 years, >10 years) seems to be protective against DED in our cohort (OR, 0.70, *p* < 0.001; OR, 0.33, *p* < 0.001) (Table 4). After adjusting for age, sex, and DM duration, we performed three different models regarding IVI (referred to model 1), TPPV (referred to model 2), and PRP (referred to model 3) as their population was highly overlapping, which caused interference if we analyzed them in the same model. As a result, IVI, PRP, TPPV, and cataract surgery were all demonstrated as risk factors for the development of DM-related DED (OR, 1.86; *p* < 0.001; OR, 2.41, *p* < 0.001; OR, 3.72, *p* < 0.001; OR, 3.81–4.16, *p* < 0.001, respectively). Higher average HbA1c level was a contributing factor in all three models (OR, 2.14, *p* < 0.001; OR, 2.14, *p* < 0.001; OR, 2.09, *p* < 0.001, respectively). For diabetic comorbidity, retinopathy (OR, 2.40, *p* < 0.001; OR, 2.22, *p* < 0.001; OR, 2.15, *p* < 0.001, respectively) and neuropathy (OR, 1.69, *p* < 0.001; OR, 1.71, *p* < 0.001; OR, 1.66, *p* < 0.001, respectively) were contributing factors in all three models. As for nephropathy, compared with those with eGFR ≥60, an eGFR between 30 and 59 was a significant risk factor in all three models (OR, 1.17, *p* < 0.001; OR, 1.17, *p* < 0.001; OR, 1.16, *p* < 0.001, respectively), although eGFR <30 was a risk factor without statistical significance. Taking anti-glycemic medications into consideration, non-routine/other medications appeared to be a protective factor against DED in all three models (OR, 0.54, *p* < 0.001, in all three models). Compared with metformin monotherapy, DPP4 inhibitor, GLP-1 agonist, SGLT-2 inhibitor, and insulin monotherapy demonstrated as protective factors for DED and dual therapy. When adopting OR as a predictor for the efficacy of preventing DED, SLGT-2 inhibitor had lower OR in all three models (OR, 0.09, *p* < 0.001; OR, 0.09, *p* < 0.001; OR, 0.09, *p* < 0.001, respectively), followed by GLP-1 agonist (OR, 0.14, *p* < 0.001; OR, 0.13, *p* < 0.001; OR, 0.14, *p* < 0.001, respectively), DPP-4 inhibitor (OR, 0.54, *p* < 0.001; OR, 0.54, *p* < 0.001; OR, 0.54, *p* < 0.001, respectively), and insulin alone (OR, 0.71, *p* = 0.004; OR, 0.72, *p* = 0.006; OR, 0.72, *p* = 0.005, respectively). For dual therapy group, the OR between DPP4 inhibitor and GLP-1 agonist (OR, 0.33, *p* < 0.001; OR, 0.34, *p* < 0.001; OR, 0.34, *p* < 0.001, respectively) (Table 5). Given the retrospective nature of our study, without active interventions, there were no safety concern and any adverse events.

**Table 1:** Demographics and characteristics of dry eye disease (DED) and non-dry eye disease (non-DED) groups in patients with type II diabetes mellitus.

	**DED** ^*^		**Non-DED**		***P*** **-value**
	***N*** = **10,029**		***N*** = **142,491**		
**Characteristic**	***n***	**%**	***n***	**%**	
**Age**					
Age at the diagnosis of type II DM	59.41 ± 12.28		59.74 ± 13.35		0.012
Age at the end point of follow-up^*^	63.72 ± 12.41		65.12 ± 13.43		<0.001
<65 years	5,134	51.2	66,243	46.5	<0.001
≥65 years	4,895	48.8	76,248	53.5	
Sex					<0.001
Male	4,918	49.0	82,224	57.7	
Female	5,111	51.0	60,267	42.3	
**HbA1c (M ±SD)**					
First data	8.45 ± 2.45		8.35 ± 2.43		<0.001
Mean data	7.76 ± 1.64		7.67 ± 1.55		<0.001
**Type II DM duration**					
Mean	4.27 ± 3.52		5.45 ± 4.26		<0.001
<5 years	6,481	64.6	76,213	53.5	
5–10 years	2,662	26.5	40,648	28.5	
≥10 years	886	8.8	25,630	18.0	
**Comorbidity**					
Retinopathy	2,124	21.2	11,231	7.9	<0.001
Neuropathy	1,587	15.8	15,991	11.2	<0.001
Nephropathy	2,239	22.3	33,676	23.6	0.003
egfr					<0.001
≥60	4,957	49.4	68,877	48.3	
30–59	2,581	25.7	35,926	25.2	
<30	2,491	24.8	37,688	26.5	

* DED, dry eye disease.

*Age at the end point of follow-up: In the DED group, it is the time point of patients having the diagnosis of dry eye disease; in the non-DED group, it is the time point of the last follow-up.

**Table 2:** Demographics and characteristics of the dry eye disease and non-dry eye disease groups in patients with type II diabetes mellitus after age and sex match.

	**DED** ^*^		**Non-DED**		***P*** **-value**
	***N*** = **9,541**		***N*** = **38,164**		
**Characteristic**	***n***	**%**	***n***	**%**	
**Age**					
Age at the diagnosis of type II DM	59.4 ± 12.3		59.2 ± 12.3		0.299
Age at the end point of follow-Up^*^	63.7 ± 12.4		63.7 ± 12.4	1.000
<65 Years old	4,885	51.2	19,540	50.2	1.000
<65 Years old	4,652	48.8	18,608	48.8	
Sex			1.000		
Male	4,789	50.2	19,156	50.2	
Female	4,748	49.8	18,992	49.8	
**HbA1c (M ±SD)**					
First data	8.5 ± 2.5		8.3 ± 2.4		<0.001
mean data	7.8 ± 1.6		7.6 ± 1.5		<0.001
**Type II DM duration**					
<5 years	6,039	63.3	24,156	63.3	1.000
5–10 years	2,620	27.5	10,480	27.5	
≥ 10 years	878	9.2	3,512	9.2	
**Comorbidity**					
Retinopathy	2,020	21.2	2,912	7.6	<0.001
Neuropathy	1,539	16.1	3,372	8.8	<0.001
Nephropathy	2,146	22.5	8,622	22.6	0.835
egfr					<0.001
≥60	4,680	49.1	20,517	53.8	
30–59	2,478	26.0	8,891	23.3	
<30	2,379	24.9	8,740	22.9	

* DED, dry eye disease.

*Age at the end point of follow-up: In the DED group, it is the time point of patients having the diagnosis of dry eye disease; in the non-DED group, it is the time point of the last follow-up.

**Table 3:** Hypoglycemic medications used and procedures received in both dry eye disease and non-dry eye disease groups after age and sex match.

	**DED** ^*^		**Non-DED**		***P*** **-value**
	***N*** **= 9,541**		***N*** **= 38,164**		
	***n***	**%**	***n***	**%**	
**Medication**					
Other/non-routine medications^*^	5,536	58.3	22,885	60.0	<0.001
**Routine medication**					
Metformin	2,000	21.0	4,848	12.7	
DPP4 inhibitor	553	5.8	1,945	5.1	
GLP-1 agonist	5	0.1	399	0.2	
SGLT-2 inhibitor	18	0.2	62	1.1	
Insulin	123	1.3	311	0.8	
Other dual medications^*^	1,275	14.1	7,698	20.1	
**Procedure**					
IVI^*^	583	6.1	566	1.5	<0.001
TPPV^*^	684	7.2	323	0.9	<0.001
Cataract surgery	1,231	12.9	1,065	2.8	<0.001
PRP^*^	874	9.2	701	1.8	<0.001

* Other/non-routine medications: medications other than metformin, DPP4 inhibitor, GLP-1 agonist, SGLT-2 inhibitor, and insulin, and these medications that did not allow achievement of the target MPR.

*Other dual medications: DPP4 inhibitor+ insulin, DPP4 inhibitor or insulin+GLP-1 agonist, and DPP4 inhibitor or insulin+ SGLT-2 inhibitor.

*IVI, intravitreal injection.

*TPPV, trans-pars plana vitrectomy.

*PRP, pan-retinal photocoagulation.

**Table 4:** Risk and protective factor analysis using conditional logistic regression model assessing the influence of age, sex, and DM duration on DED.

	**Adjusted OR**	**95% CI**	***P*** **-value**
**Sex**				
Male	1.00			
Female	1.02	0.97	1.07	0.390
**Age (years)**				
<65	1.00			
≥65	1.18	1.13	1.24	<0.001
**Type II DM duration**				
<5 years	1.00			
5–10 years	0.70	0.67	0.74	<0.001
≥10 years	0.33	0.31	0.36	<0.001

**Table 5:** Risk and protective factor analysis for dry eye disease using the conditional logistic regression model with control of age, sex, and DM.

	**Adjust OR**	**95% CI**		***P*** -value 1^*^	**Adjust OR**	**95% CI**		***P*** -value 2^*^	**Adjust OR**	**95% CI**		***P*** -value 3^*^
Mean HbA1c	2.14	1.83	2.50	<0.001	2.14	1.83	2.51	<0.001	2.09	1.79	2.44	<0.001
**Medication**												
Other/non-routine medications^*^	0.54	0.51	0.57	<0.001	0.54	0.50	0.57	<0.001	0.54	0.51	0.58	<0.001
**Routine medication**												
Metformin	1.00				1.00				1.00			
DPP4 inhibitor	0.54	0.48	0.61	<0.001	0.54	0.48	0.61	<0.001	0.54	0.48	0.60	<0.001
GLP-1 agonist	0.14	0.05	0.36	<0.001	0.13	0.05	0.34	<0.001	0.14	0.05	0.35	<0.001
SGLT-2 inhibitor	0.09	0.05	0.15	<0.001	0.09	0.05	0.15	<0.001	0.09	0.05	0.15	<0.001
Insulin	0.71	0.56	0.89	0.004	0.72	0.57	0.91	0.006	0.72	0.57	0.91	0.005
Other dual medications^*^	0.33	0.31	0.36	<0.001	0.34	0.31	0.37	<0.001	0.34	0.31	0.36	<0.001
**Comorbidity**												
Neuropathy	1.69	1.57	1.82	<0.001	1.71	1.59	1.84	<0.001	1.66	1.55	1.78	<0.001
Retinopathy	2.40	2.23	2.58	<0.001	2.22	2.06	2.38	<0.001	2.15	1.99	2.32	<0.001
**Nephropathy (eGFR)**												
≧60	1.00				1.00				1.00			
30–59	1.17	1.11	1.25	<0.001	1.17	1.10	1.24	<0.001	1.16	1.09	1.23	<0.001
<30	1.04	0.98	1.11	0.181	1.03	0.96	1.06	0.441	1.02	0.96	1.09	0.561
**Procedure**												
IVI^*^	1.86	1.62	2.13	<0.001								
TPPV^*^					3.72	3.19	4.34	<0.001				
PRP^*^									2.42	2.14	2.73	<0.001
Cataract surgery	4.16	3.78	4.57	<0.001	3.81	3.46	4.20	<0.001	4.13	3.75	4.54	<0.001

* P-value 1: adjusted by sex, age, DM duration, neuropathy, retinopathy, DM drugs, eGFR, IVI, and cataract surgery.

*P-value 2: adjusted by sex, age, DM duration, neuropathy, retinopathy, DM drugs, eGFR, TPPV, and cataract surgery.

*P-value 3: adjusted by sex, age, DM duration, neuropathy, retinopathy, DM drugs, eGFR, PRP, and cataract surgery.

*Other/non-routine medications: medications other than metformin, DPP4 inhibitor, GLP-1 agonist, SGLT-2 inhibitor, and insulin and these medications that did not allow achievement of the target MPR.

*Other dual medications: DPP4 inhibitor+ insulin, DPP4 inhibitor or insulin+ GLP-1 agonist, and DPP4 inhibitor or insulin+ SGLT-2 inhibitor.

*IVI, intravitreal injection.

*TPPV, trans-pars plana vitrectomy.

*PRP, pan-retinal photocoagulation.

## Discussion

To the best of our knowledge, this is the first study to investigate the association between DED and antihyperglycemic agent, and we found that DPP4 inhibitor, GLP-1 agonist, SGLT-2 inhibitor, and insulin monotherapy are all superior to metformin alone. In terms of predicting the protective effect using OR, SGLT-2 inhibitor is the highest, followed by GLP-1 agonist, DPP4 inhibitor, and insulin. As for the protective effect of dual medications, due to insufficient case number, all individuals using dual medications are pooled together for analysis, and thus it is difficult for us to identify the effect of different combinations. However, the combined protective effect between DPP4 inhibitor and GLP-1 agonist was observed. In our study, we also verified different possible risk factors for DM-related DED and found consistent results compared with previous studies (3, 4, 16, 28, 29), including female sex, advanced age, poor glycemic control evidenced by higher average HbA1c level, and presence of diabetic retinopathy. For diabetic neuropathy and nephropathy, controversial results were shown among different studies (26, 30, 31). In our cohort, neuropathy was demonstrated as a risk factor for DED in the DM population, and the risk increased with the deterioration of renal function (eGFR) in DM nephropathy, which was consistent with the results of a previous study (31).

An intriguing finding is that longer DM duration appeared to be a protective factor for the development of DED in our study. In previous studies, DM duration either did not have a significant effect or is a contributing factor for DED (32, 33). However, studies had discovered that, in patients with longer DM duration, self-reported symptoms and decreased corneal sensitivity along with inferior whorl length destruction were noted with high underdiagnosis rate for DED (8, 34). Therefore, in a retrospective database study, a proportion of underdiagnosed individuals were anticipated as patients would not seek for medical help due to minimal symptom, especially in those with long-term DM. In our study, when using CGRD rather than the national health insurance database, retrieving laboratory data became possible. However, if the patients visited ophthalmologists elsewhere and had been diagnosed with DED, these patients will be categorized as non-DED group in CGRD database. According to the criteria we set to define the DM duration of the non-DED group, the DM duration of these patients would not meet its endpoint until the last follow-up date in our hospital system rather than the day they had been diagnosed with DED; thus, the duration in the non-DED group might be prolonged. To deal with this bizarre scenario, we had adjusted the influence of DM duration in our regression model for other risk factor analyses.

DED is a multifactorial disease, in which inflammation and neurosensory abnormalities are involved in its pathogenesis (9); moreover, lacrimal functional unit dysfunction is known to be related to diabetic neuropathy, which leads to the development of DM-related DED (14, 15). The neuroprotective and anti-inflammatory effect of DPP4 inhibitor, SGLT-2 inhibitor, and GLP-1 agonist had been investigated in previous studies (35). For instance, SGLT-2 inhibitors have a dose-dependent effect on diabetic neuropathy in mice (36), and its ability to reduce oxidative stress and inflammation had also been reported (37). As for GLP-1 agonist, a preclinical trial had shown beneficial effects on diabetic polyneuropathy and peripheral nerve degeneration in human and neuroprotective effect in animal models (38). In DPP-4 inhibitors, different studies on diabetic rats had pointed out its ability to reduce the decrease in nerve density, restoring mechanical sensitivity thresholds, and improving nerve conduction velocity and slowing nerve fiber atrophy (39–41). In humans, DPP-4 inhibitor was found to be superior to sulfonylurea drugs in preventing diabetic neuropathy (42). In contrast, metformin was found to be associated with more severe diabetic peripheral neuropathy compared with non-metformin treatment (43). From the results of our study, all GLP-1 agonists, SGLT-2 inhibitors, and DPP-4 inhibitors are superior to metformin alone in preventing DM-related DED. We presume that the possible mechanisms contributing to the results are the neuroprotective effect against DM neuropathy as demonstrated by these medications, which further prevents the occurrence of lacrimal function unit dysfunction, resulting in lower incidence of DM-related DED in patients using antihyperglycemic agents.

There are several limitations in this study. First, when using CGRD database, we could not investigate the integral follow-up course for some patients as a few of them might have visited other institutions, which might cause bias regarding our grouping. Second, the CGRD does not contain diagnostic data for DED including Schirmer test and ocular surface disease index questionnaire; therefore, we can only define DED using ICD diagnostic code combined with medications used. Moreover, most patients are Asian; thus, the result of our study may need further validation to be applicable to other ethnicities. Lastly, due to the retrospective nature of our study, despite the strict inclusion and exclusion criteria, there might still be some bias regarding grouping and accuracy of diagnosis.

## Conclusion

DED in patients with DM are associated with female sex, advanced age, poor glycemic control, and development of comorbidities. Ocular procedures, including IVI, PRP, TPPV, and cataract surgery, also increase the risk of developing DED. However, in our cohort, longer DM duration appeared to be protective against DED, which might be masked due to underdiagnosis caused by peripheral neuropathy. As for antihyperglycemic agent, GLP-1 agonist, SGLT-2 inhibitor, DPP4 inhibitor, and insulin monotherapy are all superior to metformin alone, with SGLT2 inhibitor and GLP-1 agonist having significantly lower OR, followed by DPP-4 inhibitor and insulin. However, a prospective randomized control trial will be needed to further consolidate our results.

## Data availability statement

The original contributions presented in the study are included in the article/Supplementary material, further inquiries can be directed to the corresponding author.

## Ethics statement

The studies involving human participants were reviewed and approved by Review Board of Chang Gung Memorial Hospital (approval no.: 202001925B0C601). Written informed consent for participation was not required for this study in accordance with the national legislation and the institutional requirements.

## Author contributions

L-YP and C-CS contributed to statistical analysis and manuscript composition. Y-KK, L-YP, C-CS, and T-HC contributed to study design and data collection. All authors contributed to the article and approved the submitted version.

## Funding

The study was supported by a grant from the Alcon Services AG, Taiwan Branch (Investigator-Initiated Research IIR # 57241343).

## Conflict of interest

The authors declare that the research was conducted in the absence of any commercial or financial relationships that could be construed as a potential conflict of interest.

## Publisher's note

All claims expressed in this article are solely those of the authors and do not necessarily represent those of their affiliated organizations, or those of the publisher, the editors and the reviewers. Any product that may be evaluated in this article, or claim that may be made by its manufacturer, is not guaranteed or endorsed by the publisher.
