# Supplementary material for: Dry eye disease in patients with type II diabetes mellitus: A retrospective, population-based cohort study in Taiwan
Source: Front Med (Lausanne). 2022 Aug 23;9:980714. doi: 10.3389/fmed.2022.980714 (PMC9445241; doi:10.3389/fmed.2022.980714)
Supplement: Supplementary file 1 [file Table_1.pdf]

Supplement Table 1: ICD-9-CM and ICD-10-CM code for comorbidities

DM* retinopathy		DM nephropathy		DM neuropathy	
ICD-9-CM	ICD-10-CM	ICD-9-CM	ICD-10-CM	ICD-9-CM	ICD-10-CM
250.5x	E11.3xy	249.4x	E11.2x	249.6x	E11.4x
(x=0-3)	(x=1-5)	(x=0,1)	(x=1,2,9)	(x=0-1)	(x=0,1,2,3,9)
	(y=1.9)				
249.5x	E11.39	250.4x		250.6x	
(x=0-1)		(x=0-3)		(x=0-3)	
362.0x					
(x=1-7)					

*DM: diabetes mellitus
