# Supplementary material for: Dry eye disease in patients with type II diabetes mellitus: A retrospective, population-based cohort study in Taiwan
Source: Front Med (Lausanne). 2022 Aug 23;9:980714. doi: 10.3389/fmed.2022.980714 (PMC9445241; doi:10.3389/fmed.2022.980714)
Supplement: Supplementary file 2 [file Table_2.pdf]

Supplement Table 2: Surgical codes for ocular procedures

IVI*	TPPV*	PRP*	Cataract
65-861	86206b	S47-321	86006c
86201c	86207b	S47-323	86007c
	86208c		86008c
	86410b		86011c
	86411b		86012c
	86412b		86013c
	86413b		
	86414b		
	86415b		

*IVI: intravitreal injection

*TPPV: trans-pars plana vitrectomy

*PRP: pan-retinal photocoagulation
